# Cross-sectional study of the association between skin tags and vascular risk factors in a bariatric clinic-based cohort of Irish adults with morbid obesity

**DOI:** 10.1186/s13104-020-05006-4

**Published:** 2020-03-16

**Authors:** Clarissa Ern Hui Fang, Catherine Crowe, Annette Murphy, Martin O’Donnell, Francis M. Finucane

**Affiliations:** 1 Bariatric Medicine Service, Centre for Diabetes, Endocrinology and Metabolism, Galway University Hospitals, Galway, Ireland; 2 HRB Clinical Research Facility, National University of Ireland Galway, Galway, H91 YR71 Ireland; 3 Department of Dermatology, Galway University Hospitals, Galway, Ireland

**Keywords:** Skin tags, Acrochordons, Morbid obesity, Bariatric, Hypertension, Type 2 diabetes

## Abstract

**Objective:**

Skin tags are associated with an insulin resistant phenotype but studies in White Europeans with morbid obesity are lacking. We sought to determine whether the presence of cervical or axillary skin tags was associated with increased cardiovascular risk in Irish adults with morbid obesity. We conducted a cross-sectional study of patients attending our Irish regional bariatric centre with a BMI ≥ 40 kg m^−2^ (or ≥ 35 kg m^−2^ with co-morbidities). We compared anthropometric and metabolic characteristics in those with versus without skin tags.

**Results:**

Of 164 patients, 100 (31 male, 37 with type 2 diabetes, 36 on lipid lowering therapy, 41 on antihypertensive therapy) participated. Mean age was 53.7 ± 11.3 (range 31.1–80) years. Cervical or axillary tags were present in 85 patients. Those with tags had higher systolic blood pressure 138.0 ± 16.0 versus 125.1 ± 8.3 mmHg, p = 0.003) and HbA1c (46.5 ± 13.2 versus 36.8 ± 3.5 mmol/mol, p = 0.017). Tags were present in 94.6% of patients with diabetes, compared to 79.4% of those without diabetes (p = 0.039). Antihypertensive therapy was used by 45.8% of patients with skin tags compared to 13.3% without tags (p = 0.018). In bariatric clinic attenders skin tags were associated with higher SBP and HbA1c and a higher prevalence of diabetes and hypertension, consistent with increased vascular risk, but lipid profiles were similar.

## Introduction

Obesity is known to cause increased mortality [1] and morbidity [2] from diseases such as type 2 diabetes. However, there is heterogeneity in the relationship between excess body fat and disease risk. For example, some patients develop diabetes at a relatively low level of adiposity while others never develop diabetes, even with morbid obesity [3]. Insulin resistance, the key defect linking obesity and type 2 diabetes [4], also drives increased vascular risk [5]. However precise measurement of insulin resistance in routine clinical practice is usually not feasible [6]. Cutaneous markers of insulin resistance include acanthosis nigricans, androgenetic alopecia, acne, hirsutism and skin tags (acrochordons) [6–8]. These benign, pedunculated papular lesions are commonly located on the neck, axillae, eyelids and groin. While their association with diabetes has long been recognized [9], skin tags are not usually considered in the clinical assessment of patients or emphasized in clinical practice guidelines and, rather like insulin resistance itself, they represent an abstract academic consideration at the patient’s bedside rather than a relevant and quantifiable indicator of vascular risk. Nonetheless, in our clinical practice (in a regional bariatric centre in the West of Ireland) we have found skin tags to be a useful marker of insulin resistance in patients presenting with atypical diabetes phenotypes [10, 11].

Skin tags are thought to arise from high levels of insulin and associated growth factors causing proliferation of epidermal fibroblasts [6]. They have been associated with various indicators of increased vascular risk including an adverse lipid profile [12–16], insulin resistance [17, 18], impaired glucose metabolism [19, 20], elevated blood pressure [14, 21] and components of the metabolic syndrome [7, 14, 21–23]. However all of these studies have been done in non-white ethnic groups or those without morbid obesity. Specifically, the clinical significance of the presence of skin tags in White European patients with morbid obesity has not been determined previously. Also, the extent to which the presence of skin tags predicts an adverse cardiovascular risk profile in this group is not clear. We sought to determine whether skin tags were associated with an adverse vascular risk profile in a predominantly white population of Irish adults attending our bariatric centre for management of morbid obesity.

## Main text

### Patients and methods

This was a single centre cross-sectional study. The study population included a convenience sample of patients ≥ 18 years old with a BMI (body mass index) ≥ 40 kg m^−2^ (or ≥ 35 with co-morbidity) who were attending a hospital-based bariatric outpatient department. We excluded patients who were pregnant or unable to provide informed consent. The local research ethics committee approved the study (C.A. 952) on 19th July 2013. We reported the numbers of patients approached and recruited in line with STROBE guidelines [24]. At assessment, we obtained fully informed written consent from each participant. We noted the use of cardioprotective medications such as antihypertensive and lipid lowering drugs. Weight was measured using a Seca^®^ scale and height with a Seca^®^ Leicester stadiometer. Systolic blood pressure (SBP) and diastolic blood pressure were measured with an Omron^®^ 705IT oscillometric device in the left arm after the patient was sitting comfortably for 5 min. Ethnicity was self-reported.

### Laboratory analysis

We analysed all blood samples locally in the Galway University Hospitals’ Department of Clinical Biochemistry (certified to ISO 15189 2007 accreditation standard). HbA1c was measured with high performance liquid chromatography (Menarini^®^ HA8160 auto-analyzer). Total cholesterol was measured using the CHOP-PAP method. High density lipoprotein (HDL)-cholesterol and triglycerides were measured using the enzymatic and the GPO-PAP methods, respectively (Roche COBAS^®^ 8000 modular analyzer). Low density lipoprotein (LDL)-Cholesterol was derived with the Friedewald equation [25]. We derived the triglyceride:HDL-cholesterol ratio (THDLR) as a surrogate marker of insulin resistance [26].

### Dermatological assessment

A single investigator (CC) conducted all patient dermatological assessments having undergone a period of clinical instruction in acrochordon assessment by a consultant dermatologist (AM) and according to a standard operating procedure. Each participant was given the opportunity to have a chaperone present for the examination. Where acrochordons were present, their location (axillary or cervical), number and size were documented. We did not examine patients for the presence of inframammary or groin skin tags, as we felt that this would not be relevant to routine bariatric clinical assessment.

### Statistical analysis

We determined the differences between participants with versus those without skin tags using the unpaired t-test and differences in proportions were assessed with the Chi-square test. Where we found differences in those with versus without skin tags, we assessed the influence of confounders such as medication usage, sex, age and BMI using binomial logistic regression with the presence of any skin tags being the response variable. We used linear- as well as ordinal-regression to determine whether the number of skin tags (either as a continuous or categorical dependent variable) was associated with vascular risk factors. We chose “no skin tags” as the reference category. A p-value of < 0.05 was deemed to be statistically significant. All analyses were performed using SPSS version 24.

### Results

Between December 2012 and June 2014, 164 patients attended our facility and of these, 100 agreed to participate in the study. The male: female ratio was 1:2.2. There was one patient of Black African descent, five of Irish Traveler descent and 94 of White European descent. The mean age was 53.7 ± 11.3 (range 31.1–80) years. The mean number of skin tags was 10.5 ± 18.4 (range 0–135). Tags were present in 85 patients and absent in 15. Skin tags were present only in the axillae in 30 patients, only on the neck in 11 patients and 44 patients had skin tags in both the axillae and the neck. 62 patients had three or more skin tags while 38 had less than three. 12 patients were current smokers while 88 were previous or never smokers. 14 patients reported a current or past history of sleep apnea while 4 had previous bariatric surgery. 41 patients were currently taking antihypertensive therapy (59 were not), 37 were taking medication for type 2 diabetes (none had type 1 diabetes) and 36 patients were taking lipid lowering therapy (either statin or fibrate). Of the 37 patients with diabetes, 35 had skin tags (94.6%), compared to 50 of 63 patients without diabetes (79.4%, p = 0.039). Put another way, 59% of those with skin tags, compared to 13% of those with no tags had diabetes. Skin tags were present in 79.7% of female and 96.8% of male patients (p = 0.027). 45.8% of patients with skin tags compared to 13.3% with no tags were on antihypertensive therapy (p = 0.018), while 38.8% and 20%, respectively, were on lipid lowering therapy (p = 0.16).

The anthropometric and metabolic characteristics of study participants with any skin tags compared to those with no skin tags are presented in Table 1. Those with any skin tags had higher SBP and fasting blood glucose and a higher HbA1c. There was a (statistically non-significant) trend to higher THDLR in those with skin tags, consistent with them being more insulin resistant. Likewise, there were non-significant trends to increased weight and diastolic blood pressure in those with versus those without any skin tags.

**Table 1:** Baseline characteristics and clinical measurements of adults with morbid obesity comparing those with either axillary or cervical skin tags to those with no skin tags

Variable	No skin tags		Any skin tags		p-value
	n	15	n	85	
Weight (kg)	122.1	± 15.3	130.2	± 27.4	0.113
Height (m)	1.61	± 0.07	1.68	± 0.11	0.007^#^
Waist circumference (cm)	133.7	± 12.8	136.3	± 18.9	0.611
BMI (kg/m^2^)	47.1	± 6.6	46	± 7.8	0.61
Systolic blood pressure (mmHg)	125.1	± 8.3	138.0	± 16.0	< 0.001^#^
Diastolic blood pressure (mmHg)	80.4	± 9.7	85.6	± 9.7	0.094
Total cholesterol (mmol/L)	5	± 1.0	4.6	± 1.2	0.19
LDL cholesterol (mmol/L)	3.1	± 0.9	2.6	± 1.2	0.136
HDL cholesterol (mmol/L)	1.3	± 0.3	1.2	± 0.3	0.329
Triglycerides (mmol/L)	1.5	± 0.7	1.8	± 1.1	0.351
Triglyceride:HDL	1.3	± 0.6	1.7	± 1.4	0.054
total cholesterol (mmol/L)^c^	5.3	± 0.8	4.8	± 1.2	0.155
LDL cholesterol (mmol/L)^c^	3.4	± 0.7	2.8	± 1.1	0.103
HDL cholesterol (mmol/L)^c^	1.2	± 0.2	1.2	± 0.3	0.993
Triglycerides (mmol/L)^c^	1.6	± 0.7	1.7	± 1.0	0.712
Triglyceride:HDL^c^	1.3	± 0.6	1.6	± 1.4	0.543
Total cholesterol (mmol/L)^d^	3.9	± 0.8	4.2	± 1.3	0.666
LDL cholesterol (mmol/L)^d^	1.9	± 0.3	2.3	± 1.2	0.587
HDL cholesterol (mmol/L)^d^	1.5	± 0.7	1.1	± 0.3	0.128
Triglycerides (mmol/L)^d^	1.1	± 0.3	1.9	± 1.2	0.288
Triglyceride:HDL ratio^d^	0.9	± 0.5	1.8	± 1.5	0.306
Glucose (mmol)	5.1	± 0.5	6.6	± 2.5	< 0.001^#^
HbA1c (mmol/mol)	37.8	± 3.5	46.5	± 13.2	< 0.001^#^
Glucose (mmol)^a^	5	± 0.5	5.7	± 1.9	0.189
HbA1c (mmol/mol)^a^	36.8	± 2.2	40.7	± 10.5	0.201
Glucose (mmol)^b^	5.6	± 0.4	7.9	± 2.7	0.244
HbA1c (mmol/mol)^b^	44	± 4.2	53.9	± 12.7	0.289
Functional capacity (MET max)	6	± 1.7	6.1	± 2.0	0.859

Data are presented as means ± standard deviations

^a^Denotes subgroup without diabetes (no skin tags n = 13, skin tags n = 50)

^b^Denotes subgroup with diabetes (no skin tags n = 2, skin tags n = 35)

^c^Denotes subgroup not on lipid lowering agent (no skin tags n = 12, skin tags n = 52)

^d^Denotes subgroup on lipid lowering agent (no skin tags n = 3, skin tags n = 33)

^#^p-value < 0.05

Results from logistic regression analyses, with the presence or absence of any skin tags as the binary dependent variable are shown in Table 2. For every rise of 1 mmHg in SBP, the likelihood of having any skin tags increased by 7.5% (p = 0.005) while patients with hypertension were 5.5 times more likely to have skin tags than patients with normal SBP (p = 0.031). However, the association with hypertension was no longer significant after adjusting for age and sex, both of which had a borderline significant association with skin tag presence, as shown in Table 2. There were non-significant trends to men having a 7.6 times greater likelihood than women to have skin tags (p = 0.055) while for every year older, there was a non-significant trend to a 5.1% increase in the likelihood of skin tags (p = 0.067). Similarly, there was a non-significant trend to a 4.55-fold increased likelihood of any skin tags in patients with diabetes compared to those without diabetes (p = 0.055). Those with tags had a higher HbA1c, but not after adjusting for age and sex. The borderline difference observed in the THDLR using the unpaired t-test was not replicated using logistic regression in unadjusted or adjusted analyses.

**Table 2:** Logistic regression model comparing the influence of various clinical features on the likelihood of having any skin tags versus none, in patients with morbid obesity

Variable	Beta (logit)	S.E.	Exp (Beta)	95% CI	p-value
Sex	2.033	1.060	7.636	[0.957, 60.939]	0.055
Age	0.05	0.027	1.051	[0.997, 1.109]	0.067
Diabetes status	1.515	0.791	4.55	[0.966, 21.439]	0.055
Diabetes status^a^	1.357	0.808	3.883	[0.797, 18.906]	0.093
Hypertension status	1.707	0.790	5.511	[1.171, 25.929]	0.031^#^
Hypertension status^a^	1.316	0.822	3.728	[0.745, 18.666]	0.109
SBP	0.073	0.026	1.075	[1.022, 1.132]	0.005^#^
SBP^a^	0.064	0.026	1.066	[1.013, 1.122]	0.014^#^
HbA1c	0.107	0.05	1.113	[1.01, 1.227]	0.031^#^
HbA1c^a^	0.082	0.048	1.086	[0.988, 1.194]	0.089
THDLR	0.376	0.338	1.457	[0.752, 2.824]	0.265
THDLR^a^	0.271	0.313	1.311	[0.71, 2.418]	0.387

Beta is the logit or estimated log odds of having any skin tags for every one unit rise in the variable measure

Exp (B) is the exponential of Beta. (So for example for every mmHg rise in systolic blood pressure, there is a 7.5% increased likelihood of having skin tags)

*S.E* standard error for Beta, *CI* confidence interval, *SBP* systolic blood pressure, *THDLR* triglyceride: HDL-cholesterol ratio

^a^Adjusted for age and sex

^#^p-value < 0.05

In linear regression models with the number of skin tags being the dependent or outcome variable, male sex was strongly associated with skin tags (β = 15.85 [8.61, 23.09], p < 0.001). In other words, men had approximately 16 more skin tags than women. There was no statistically significant association between age and skin tags (β = 0.32 [− 0.003, 0.63], p = 0.052).

Next, we compared anthropometric and metabolic characteristics in participants with both cervical and axillary skin tags to those who had no skin tags (Additional file 1: Table S1). As before, there were significant differences in glucose and HbA1c, but in addition to SBP, diastolic blood pressure was also elevated and there was a significant difference in weight of 14.8 kg in those with axillary and cervical tags compared to those with none. We compared patients who had axillary skin tags versus those without axillary skin tags, with the only significant difference being greater height and weight in those with axillary skin tags (Additional file 1: Table S2). Finally, we found that compared to patients without cervical skin tags, those with cervical skin tags had higher systolic and diastolic blood pressure, with higher BMI and a (borderline significant) higher waist circumference and weight (Additional file 1: Table S3).

### Discussion

In a predominantly White European cohort of Irish adults with morbid obesity, skin tags were more prevalent in patients with diabetes and hypertension and were associated with higher SBP and HbA1c. However, there were no differences in lipid profiles and the trend to an increased triglyceride:HDL cholesterol ratio in those with skin tags, suggestive of insulin resistance, did not reach statistical significance.

The population is predominantly White European, so while it limits the generalizability of the results to other ethnic groups, it also reduces random error from variations in hereditary influences on the relationship between skin tags and vascular risk factors. Given that a specially trained single operator (CC) carried out the dermatological examination according to a standard protocol and under expert dermatological guidance (AM), we have minimized information bias and inter-observer subjectivity in acrochordon assessment.

We found skin tags in 85 (85%) of our cohort compared to 236 (52.4%) in a Turkish study looking at cutaneous manifestations in obese participants [27]. Our findings of elevated blood glucose and HbA1c in patients with skin tags are consistent with observations in Indian [19, 21] and Turkish [20] cohorts. Similarly, Iranian [18] and Egyptian [17] cohorts have described impaired insulin sensitivity in those with skin tags. However the differences in the various components of the lipid profile were not more apparent in our study. Four different Indian studies [12, 13, 16, 23] and one from Turkey [14] have found atherogenic lipid profiles associated with skin tags.

Our findings need to be retested in larger studies. The utility of skin tags as a meaningful sign of insulin resistance and increased vascular risk remains to be determined. Indeed, we have not sought to quantify directly insulin resistance in this study, though this would clearly have been valuable. Currently, insulin resistance is an abstract and poorly defined state in routine clinical practice, relying to some extent on the physician’s “gestalt”. Hence there is a need to identify and validate novel surrogate clinical and metabolic measures of insulin sensitivity and vascular risk, such as has already been done with waist circumference [28] and adipokines [29], respectively.

### Conclusion

In summary our findings are novel in the context of skin tags being associated with an adverse cardiovascular risk profile in Irish adults with morbid obesity. Further studies comparing differences in insulin sensitivity and cardiovascular risk in patients with skin tags seem warranted.

## Limitations

The sample size was determined by a convenience sample of patients attending our bariatric service over 18 months, and we cannot assume that our results are generalizable to adults with morbid obesity not attending hospital bariatric services—there may have been some selection bias in the population studied. We did not collect data on the type of diabetes medications and these may have influenced THDLR. A larger number of patients would be helpful in more precisely elucidating the influence of sex, age and ethnicity on the relationship between tags and adverse phenotype.

## Supplementary information

**Additional file 1: Table S1.** Anthropometric and metabolic characteristics for adults comparing both axillary and cervical skin tags versus none. **Table S2.** Anthropometric and metabolic characteristics for adults comparing axillary skin tags versus those with no axillary skin tags. **Table S3.** Anthropometric and metabolic characteristics for adults comparing cervical skin tags versus those with no cervical skin tags.

## Data Availability

Data analysed in this study are included in this published article and its Additional file.

## Abbreviations

HbA1c: Glycated haemoglobin
BMI: Body mass index
SBP: Systolic blood pressure
HDL: High density lipoprotein
LDL: Low density lipoprotein
THDLR: Triglyceride: HDL-cholesterol ratio

## References

[1] Abramowitz MK, Hall CB, Amodu A, Sharma D, Androga L, Hawkins M (2018). Muscle mass, BMI, and mortality among adults in the United States: a population-based cohort study. PLoS ONE.

[2] Laaksonen MA, Knekt P, Rissanen H, Harkanen T, Virtala E, Marniemi J (2010). The relative importance of modifiable potential risk factors of type 2 diabetes: a meta-analysis of two cohorts. Eur J Epidemiol.

[3] Stefan N, Haring HU, Hu FB, Schulze MB (2013). Metabolically healthy obesity: epidemiology, mechanisms, and clinical implications. Lancet Diabetes Endocrinol.

[4] Shulman GI (2014). Ectopic fat in insulin resistance, dyslipidemia, and cardiometabolic disease. N Engl J Med.

[5] Yip J, Facchini FS, Reaven GM (1998). Resistance to insulin-mediated glucose disposal as a predictor of cardiovascular disease. J Clin Endocrinol Metab.

[6] Gonzalez-Saldivar G, Rodriguez-Gutierrez R, Ocampo-Candiani J, Gonzalez-Gonzalez JG, Gomez-Flores M (2017). Skin manifestations of insulin resistance: from a biochemical stance to a clinical diagnosis and management. Dermatol Ther.

[7] Karadag AS, Ozlu E, Lavery MJ (2018). Cutaneous manifestations of diabetes mellitus and the metabolic syndrome. Clin Dermatol.

[8] Boza JC, Trindade EN, Peruzzo J, Sachett L, Rech L, Cestari TF (2012). Skin manifestations of obesity: a comparative study. J Eur Acad Dermatol Venereol.

[9] Kahana M, Grossman E, Feinstein A, Ronnen M, Cohen M, Millet MS (1987). Skin tags: a cutaneous marker for diabetes mellitus. Acta Dermato-Venereologica.

[10] Hui Fang CE, Rafey MF, Cunningham A, Dinneen SF, Finucane FM (2018). Risperidone-induced type 2 diabetes presenting with diabetic ketoacidosis. Endocrinol Diabetes Metab Case Rep.

[11] Small C, Egan AM, Elhadi EM, O’Reilly MW, Cunningham A, Finucane FM (2017). Diabetic ketoacidosis: a challenging diabetes phenotype. Endocrinol Diabetes Metab Case Rep.

[12] Wali V, Wali VV (2016). Assessment of various biochemical parameters and BMI in patients with skin tags. J Clin Diagn Res.

[13] Idris S, Sunitha S (2014). Assessment of BMI, serum leptin levels and lipid profile in patients with skin tags. J Clin Diagn Res.

[14] Akpinar F, Dervis E (2012). Association between acrochordons and the components of metabolic syndrome. Eur J Dermatol.

[15] Senel E, Salmanoglu M, Solmazgul E, Bercik Inal B (2011). Acrochordons as a cutaneous sign of impaired carbohydrate metabolism, hyperlipidemia, liver enzyme abnormalities and hypertension: a case-control study. J Eur Acad Dermatol Venereol.

[16] Gorpelioglu C, Erdal E, Ardicoglu Y, Adam B, Sarifakioglu E (2009). Serum leptin, atherogenic lipids and glucose levels in patients with skin tags. Indian J Dermatol.

[17] Shaheen MA, Abdel Fattah NS, Sayed YA, Saad AA (2012). Assessment of serum leptin, insulin resistance and metabolic syndrome in patients with skin tags. J Eur Acad Dermatol Venereol.

[18] Jowkar F, Fallahi A, Namazi MR (2010). Is there any relation between serum insulin and insulin-like growth factor-I in non-diabetic patients with skin tag?. J Eur Acad Dermatol Venereol.

[19] Rasi A, Soltani-Arabshahi R, Shahbazi N (2007). Skin tag as a cutaneous marker for impaired carbohydrate metabolism: a case-control study. Int J Dermatol.

[20] Demir S, Demir Y (2002). Acrochordon and impaired carbohydrate metabolism. Acta Diabetol.

[21] Shah R, Jindal A, Patel N (2014). Acrochordons as a cutaneous sign of metabolic syndrome: a case-control study. Ann Med Health Sci Res.

[22] Salazar MR, Carbajal HA, Espeche WG, Dulbecco CA, Aizpurua M, Marillet AG (2011). Relationships among insulin resistance, obesity, diagnosis of the metabolic syndrome and cardio-metabolic risk. Diabetes Vasc Dis Res.

[23] Tripathy T, Singh BSTP, Kar BR (2019). Association of skin tag with metabolic syndrome and its components: a case-control study from eastern India. Indian Dermatol Online J.

[24] von Elm E, Altman DG, Egger M, Pocock SJ, Gotzsche PC, Vandenbroucke JP (2007). Strengthening the reporting of observational studies in epidemiology (STROBE) statement: guidelines for reporting observational studies. BMJ.

[25] Friedewald WT, Levy RI, Fredrickson DS (1972). Estimation of the concentration of low-density lipoprotein cholesterol in plasma, without use of the preparative ultracentrifuge. Clin Chem.

[26] McLaughlin T, Reaven G, Abbasi F, Lamendola C, Saad M, Waters D (2005). Is there a simple way to identify insulin-resistant individuals at increased risk of cardiovascular disease?. Am J Cardiol.

[27] Ozlu E, Uzuncakmak TK, Takır M, Akdeniz N, Karadag AS (2018). Comparison of cutaneous manifestations in diabetic and nondiabetic obese patients: a prospective, controlled study. North Clin Istanb.

[28] Haffner SM, Stern MP, Dunn J, Mobley M, Blackwell J, Bergman RN (1990). Diminished insulin sensitivity and increased insulin response in nonobese, nondiabetic Mexican Americans. Metabolism.

[29] Finucane FM, Luan J, Wareham NJ, Sharp SJ, O’Rahilly S, Balkau B (2009). Correlation of the leptin:adiponectin ratio with measures of insulin resistance in non-diabetic individuals. Diabetologia.

