# Supplementary material for: Cross-sectional study of the association between skin tags and vascular risk factors in a bariatric clinic-based cohort of Irish adults with morbid obesity
Source: BMC Res Notes. 2020 Mar 16;13:156. doi: 10.1186/s13104-020-05006-4 (PMC7077168; doi:10.1186/s13104-020-05006-4)
Supplement: Supplementary file 1 — Additional file 1: Table S1. Anthropometric and metabolic characteristics for adults comparing both axillary and cervical skin tags versus none. Table S2. Anthropometric and metabolic characteristics for adults comparing axillary skin tags versus those with no axillary skin tags. Table S3. Anthropometric and metabolic characteristics for adults comparing cervical skin tags versus those with no cervical skin tags. [file 13104_2020_5006_MOESM1_ESM.docx]

**Additional file 1**:

“Cross-Sectional Study Of The Association Between Skin Tags And Vascular Risk Factors In A Bariatric Clinic-Based Cohort Of Irish Adults With Morbid Obesity“

Table S1: Anthropometric and metabolic characteristics for adults comparing both axillary and cervical skin tags versus none.

| **Variable** | **No Skin tags** | | **Both Axillary & Cervical Skin tags** | | | **P-value** |
| --- | --- | --- | --- | --- | --- | --- |
|  | n | 15 | | n | 44 |  |
| Systolic blood pressure (mmHg) | 125.1 | ±8.3 | | 141.1 | ±15.8 | <0.001# |
| Diastolic blood pressure (mmHg) | 80.4 | ±9.7 | | 87.6 | ±11.7 | 0.037# |
| Total Cholesterol (mmol/L) | 5 | ±1 | | 4.6 | ±1.4 | 0.353 |
| LDL Cholesterol (mmol/L) | 3.1 | ±0.9 | | 2.8 | ±1.3 | 0.376 |
| HDL Cholesterol (mmol/L) | 1.3 | ±0.3 | | 1.2 | ±0.3 | 0.281 |
| Triglycerides (mmol/L) | 1.5 | ±0.7 | | 1.7 | ±1 | 0.54 |
| Triglyceride:HDL | 1.3 | ±0.6 | | 1.7 | ±1.4 | 0.299 |
| Total Cholesterol (mmol/L)+ | 5.3 | ±0.8 | | 4.8 | ±1.2 | 0.229 |
| LDL Cholesterol (mmol/L)+ | 3.4 | ±0.7 | | 3 | ±1.2 | 0.274 |
| HDL Cholesterol (mmol/L)+ | 1.2 | ±0.2 | | 1.2 | ±0.3 | 0.794 |
| Triglycerides (mmol/L)+ | 1.6 | ±0.7 | | 1.6 | ±1 | 0.992 |
| Triglyceride:HDL ratio+ | 1.3 | ±0.6 | | 1.6 | ±1.6 | 0.613 |
| Total Cholesterol (mmol/L)++ | 3.9 | ±0.8 | | 4.4 | ±1.6 | 0.624 |
| LDL Cholesterol (mmol/L)++ | 1.9 | ±0.3 | | 2.4 | ±1.4 | 0.534 |
| HDL Cholesterol (mmol/L)++ | 1.5 | ±0.7 | | 1.1 | ±0.3 | 0.181 |
| Triglycerides (mmol/L)++ | 1.1 | ±0.3 | | 1.8 | ±0.8 | 0.171 |
| Triglyceride:HDL ratio++ | 0.9 | ±0.5 | | 1.8 | ±0.9 | 0.148 |
| Glucose (mmol) | 5.1 | ±0.5 | | 5.9 | ±1.7 | 0.007# |
| HbA1c (mmol/mol) | 37.8 | ±3.5 | | 43.6 | ±11.8 | 0.007# |
| Glucose (mmol)* | 5 | ±0.5 | | 5.4 | ±1.5 | 0.384 |
| HbA1c (mmol/mol)* | 36.8 | ±2.2 | | 39 | ±10.9 | 0.478 |
| Glucose (mmol)** | 5.6 | ±0.4 | | 6.8 | ±1.8 | 0.371 |
| HbA1c (mmol/mol)** | 44 | ±4.2 | | 50.5 | ±9.8 | 0.376 |
| Weight (kg) | 122.1 | ±15.3 | | 136.9 | ±29.1 | 0.016# |
| Height (m) | 1.61 | ±0.07 | | 1.69 | ±0.10 | 0.018# |
| Waist circumference (cm) | 133.7 | ±12.8 | | 140.2 | ±19.6 | 0.241 |
| BMI (kg/m2) | 47.1 | ±6.6 | | 48 | ±8 | 0.679 |
| Functional Capacity (MET max) | 6 | ±1.7 | | 5.9 | ±1.8 | 0.861 |

Data are presented as means ± standard deviations.

*Denotes subgroup without diabetes (No Skin tags n=13, both skin tags n=27).

**Denotes subgroup with diabetes (No Skin tags n=2, both skin tags n=17).

^+^Denotes subgroup not on lipid lowering agent (No Skin tags n=12, both skin tags n=28).

^++^Denotes subgroup on lipid lowering agent (No Skin tags n=3, both skin tags n=16).

# P-value <0.05

Table S2: Anthropometric and metabolic characteristics for adults comparing axillary skin tags versus those with no axillary skin tags

| **Variable** | **No Axillary Skin tags** | | **Axillary Skin tags** | | **P-value** |
| --- | --- | --- | --- | --- | --- |
|  | n | 26 | n | 74 |  |
| Systolic blood pressure (mmHg) | 132.9 | ±14.7 | 137.1 | ±16.1 | 0.238 |
| Diastolic blood pressure (mmHg) | 82 | ±9.0 | 85.8 | ±11.6 | 0.126 |
| Total Cholesterol (mmol/L) | 4.7 | ±1.0 | 4.6 | ±1.3 | 0.745 |
| LDL Cholesterol (mmol/L) | 2.8 | ±0.9 | 2.7 | ±1.2 | 0.762 |
| HDL Cholesterol (mmol/L) | 1.3 | ±0.3 | 1.2 | ±0.3 | 0.217 |
| Triglycerides (mmol/L) | 1.6 | ±0.9 | 1.8 | ±1.1 | 0.523 |
| Triglyceride:HDL | 1.4 | ±1.3 | 1.7 | ±1.4 | 0.412 |
| Total Cholesterol (mmol/L)^+^ | 5 | ±0.9 | 4.8 | ±1.2 | 0.599 |
| LDL Cholesterol (mmol/L)^+^ | 3.1 | ±0.8 | 2.9 | ±1.1 | 0.483 |
| HDL Cholesterol (mmol/L)^+^ | 1.3 | ±0.2 | 1.2 | ±0.3 | 0.547 |
| Triglycerides (mmol/L)^+^ | 1.5 | ±0.6 | 1.7 | ±1.0 | 0.378 |
| Triglyceride:HDL^+^ | 1.3 | ±0.6 | 1.7 | ±1.5 | 0.269 |
| Total Cholesterol (mmol/L)^++^ | 4.1 | ±0.8 | 4.2 | ±1.4 | 0.86 |
| LDL Cholesterol (mmol/L)^++^ | 2 | ±0.6 | 2.3 | ±1.2 | 0.541 |
| HDL Cholesterol (mmol/L)^++^ | 1.3 | ±0.5 | 1.1 | ±0.3 | 0.26 |
| Triglycerides (mmol/L)^++^ | 1.8 | ±1.3 | 1.8 | ±1.1 | 0.95 |
| Triglyceride:HDL^++^ | 1.8 | ±2.1 | 1.8 | ±1.2 | 0.944 |
| Glucose (mmol) | 6 | ±2.3 | 6.5 | ±2.4 | 0.399 |
| HbA1c (mmol/mol) | 43.3 | ±11.3 | 45.8 | ±13.1 | 0.409 |
| Glucose (mmol)* | 5.7 | ±2.2 | 5.5 | ±1.5 | 0.61 |
| HbA1c (mmol/mol)* | 40.4 | ±9.5 | 39.6 | ±9.6 | 0.761 |
| Glucose (mmol)** | 7 | ±2.5 | 7.9 | ±2.7 | 0.477 |
| HbA1c (mmol/mol)** | 51.8 | ±12.7 | 53.6 | ±12.8 | 0.759 |
| Weight (kg) | 121.1 | ±17.5 | 131.7 | ±28.0 | 0.028# |
| Height (m) | 1.62 | ±0.08 | 1.69 | ±0.11 | 0.002# |
| Waist circumference (cm) | 134 | ±13.7 | 136.6 | ±19.4 | 0.527 |
| BMI (kg/m^2^) | 46.5 | ±6.3 | 46 | ±8.0 | 0.791 |
| Functional Capacity (MET max) | 5.8 | ±1.7 | 6.2 | ±2.0 | 0.42 |

Data are presented as means ± standard deviations.

*Denotes subgroup without diabetes (No Skin tags n=20, axillary skin tags n=43).

**Denotes subgroup with diabetes (No Skin tags n=6, axillary skin tags n=31).

^+^ Denotes subgroup not on lipid lowering agent (No Skin tags n=17, axillary skin tags n=47).

^++^Denotes subgroup on lipid lowering agent (No Skin tags n=9, axillary skin tags n=27).

# P-value <0.05

Table S3: Anthropometric and metabolic characteristics for adults comparing cervical skin tags versus those with no cervical skin tags

| **Variable** | **No Cervical Skin tags** | | **Cervical Skin tags** | | **P-value** |
| --- | --- | --- | --- | --- | --- |
|  | n | 45 | n | 55 |  |
| Systolic blood pressure (mmHg) | 129.3 | ±13.4 | 141.6 | ±15.5 | <0.001# |
| Diastolic blood pressure (mmHg) | 82.3 | ±10.7 | 86.9 | ±11.1 | 0.04# |
| Total Cholesterol (mmol/L) | 4.7 | ±1.1 | 4.6 | ±1.3 | 0.592 |
| LDL Cholesterol (mmol/L) | 2.7 | ±1.0 | 2.7 | ±1.2 | 0.796 |
| HDL Cholesterol (mmol/L) | 1.2 | ±0.3 | 1.2 | ±0.3 | 0.639 |
| Triglycerides (mmol/L) | 1.8 | ±1.1 | 1.7 | ±1.0 | 0.662 |
| Triglyceride:HDL | 1.6 | ±1.2 | 1.7 | ±1.5 | 0.797 |
| Total Cholesterol (mmol/L)^+^ | 5 | ±1.0 | 4.7 | ±1.2 | 0.283 |
| LDL Cholesterol (mmol/L)^+^ | 3 | ±0.9 | 2.9 | ±1.2 | 0.619 |
| HDL Cholesterol (mmol/L)^+^ | 1.2 | ±0.3 | 1.2 | ±0.3 | 0.938 |
| Triglycerides (mmol/L)^+^ | 1.8 | ±0.9 | 1.5 | ±1.0 | 0.233 |
| Triglyceride:HDL^+^ | 1.6 | ±1.0 | 1.5 | ±1.5 | 0.782 |
| Total Cholesterol (mmol/L)^++^ | 4 | ±1.0 | 4.3 | ±1.4 | 0.417 |
| LDL Cholesterol (mmol/L)^++^ | 2.1 | ±0.8 | 2.4 | ±1.3 | 0.452 |
| HDL Cholesterol (mmol/L)^++^ | 1.2 | ±0.4 | 1.1 | ±0.3 | 0.65 |
| Triglycerides (mmol/L)^++^ | 1.7 | ±1.3 | 1.9 | ±1.0 | 0.527 |
| Triglyceride:HDL^++^ | 1.6 | ±1.5 | 1.9 | ±1.5 | 0.531 |
| Glucose (mmol) | 6.5 | ±2.6 | 6.2 | ±2.1 | 0.436 |
| HbA1c (mmol/mol) | 45.2 | ±12.9 | 45.1 | ±12.4 | 0.98 |
| Glucose (mmol)* | 5.4 | ±1.2 | 5.7 | ±2.0 | 0.425 |
| HbA1c (mmol/mol)* | 38.8 | ±5.8 | 40.8 | ±11.9 | 0.439 |
| Glucose (mmol)** | 8.7 | ±3.1 | 7 | ±2.0 | 0.052 |
| HbA1c (mmol/mol)** | 55.5 | ±14.8 | 51.6 | ±10.6 | 0.357 |
| Weight (kg) | 123.5 | ±22.1 | 133.5 | ±28.3 | 0.056 |
| Height (m) | 1.67 | ±0.11 | 1.67 | ±0.10 | 0.866 |
| Waist circumference (cm) | 132.2 | ±16.4 | 139 | ±18.9 | 0.063 |
| BMI (kg/m^2^) | 44.4 | ±7.2 | 47.6 | ±7.7 | 0.038# |
| Functional Capacity (MET max) | 6.4 | ±2.1 | 5.8 | ±1.7 | 0.184 |

Data are presented as means ± standard deviations.

*Denotes subgroup without diabetes (No Skin tags n=29, cervical skin tags n=34).

**Denotes subgroup with diabetes (No Skin tags n=16, cervical skin tags n=21).

^+^Denotes subgroup not on lipid lowering agent (No Skin tags n=31, cervical skin tags n=33).

^++^Denotes subgroup on lipid lowering agent (No Skin tags n=14, cervical skin tags n=22).

# P-value <0.05
